# Supplementary material for: Forgotten but not gone: A multi-state analysis of modern-day debt imprisonment
Source: PLoS One. 2023 Sep 13;18(9):e0290397. doi: 10.1371/journal.pone.0290397 (PMC10499213; doi:10.1371/journal.pone.0290397)
Supplement: S1 Appendix — (PDF) [file pone.0290397.s001.pdf]

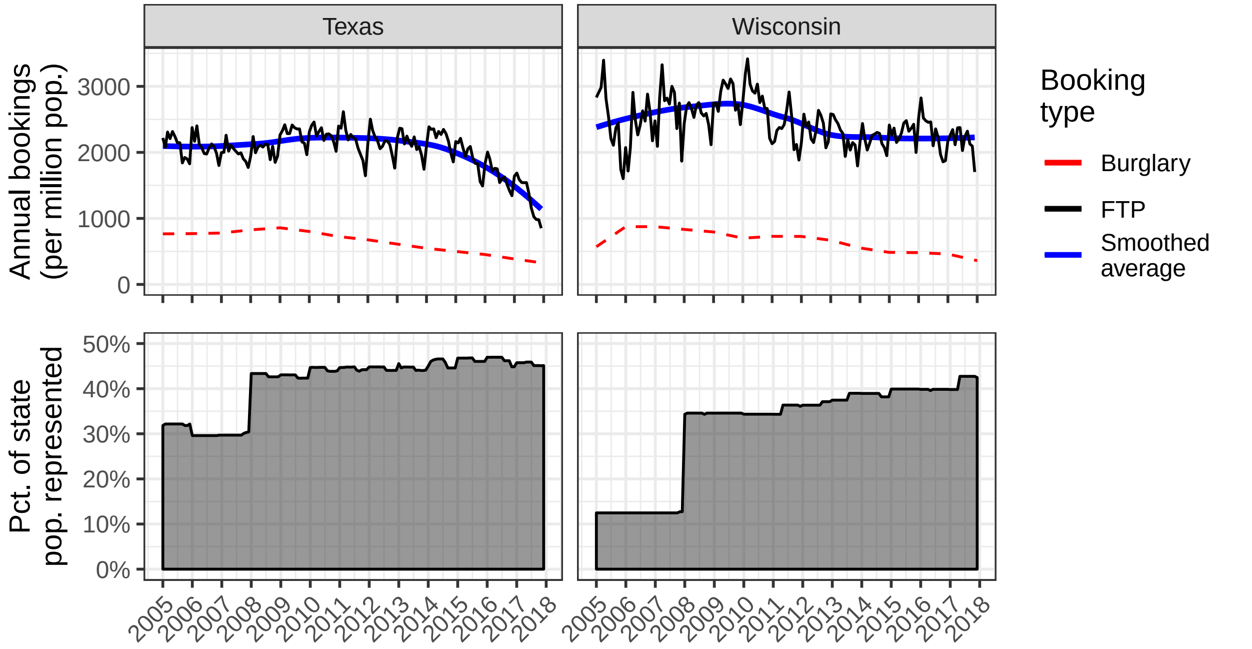

Figure S1a: ***Per capita* booking rate for individuals with *any* failure to pay charges in Texas and Wisconsin, estimated according to alternative methodology.** (Cf. Fig 2.) The top panels show the annualized *per capita* booking rates for individuals booked with *any* failure to pay charges and the bottom panels show the total population of the counties used in a given month to estimate the *per capita* rate as a percentage of the state population. The blue line in the top panels indicates a LOESS smoothed average of the annualized FTP booking rate.

S1. Robustness checks: *per capita* rates

The definition of debt imprisonment given in the main text required the exclusion of counties that did not provide the full list of charges for individuals booked in the county jail. A number of large counties—including Harris County, the largest in Texas, and Milwaukee County, the largest in Wisconsin—only provided charges related to failure to pay.

The annualized *per capita* booking rates for failure to pay (possibly among other charges) is shown in Fig S1a. This figure differs from Fig 2 only in two respects: (1) it includes bookings in which any charge makes reference to failure to pay, and (2), it includes bookings in counties where not all charges are available.

The most salient differences between Figs 2 and Fig S1a are (1) that the booking rate for individuals with *any* FTP charges is around one third higher than the booking rate for individuals with *only* FTP charges in Texas and two thirds higher in Wisconsin, and (2) these rates are calculated using counties which are home to a much larger portion of each state's citizens. (In particular, 64 counties accounting for 48.4% of Texas's population and 25 counties accounting for 45.5% of Wisconsin's population.)

In the 58 Texas and 20 Wisconsin counties in which it is possible to distinguish between individuals booked with *all* FTP charges and individuals booked with *any* FTP charges, there were approximately 80% more bookings that included any failure to pay charges than included only failure to pay charges. Assuming this ratio holds across all the counties included in Fig S1a, we can estimate that the *per capita* rate of jailings for failure to pay *alone* in these counties is around 1,100 bookings per million residents in Texas and around 1,400 bookings per million residents in Wisconsin, in relatively close agreement with our earlier estimates in the main text.
