# Supplementary material for: Forgotten but not gone: A multi-state analysis of modern-day debt imprisonment
Source: PLoS One. 2023 Sep 13;18(9):e0290397. doi: 10.1371/journal.pone.0290397 (PMC10499213; doi:10.1371/journal.pone.0290397)
Supplement: S2 Appendix — (PDF) [file pone.0290397.s002.pdf]

S4. Robustness checks: racial disparities

The analysis of racial disparities presented in the main text compares county-level demographics to the demographics of individuals jailed for failure to pay. If, however, the population of individuals jailed for failure to pay consists largely of individuals from other counties, the county-level demographics may not provide an adequate baseline. If we restrict to individuals who, when booked into jail, provided ZIP codes that match the county in question, we find that Black individuals are overrepresented among all bookings (23%) and FTP bookings (22%) relative to their share of the population living below the poverty line (10%). Roughly the same pattern holds in Wisconsin, where Black individuals make up 15% of bookings for any reason and 15% of bookings for FTP, but only 2% of the population living below the poverty line in the included counties. Hispanic individuals are represented approximately in proportion to their share of the population living below the poverty line or even underrepresented: 61% of bookings for any reason and 61% of failure to pay bookings are of Hispanic individuals in Texas, compared to 70% of the population of the included counties living below the poverty line; and 3% of all bookings and 3% of FTP bookings, compared to 4% of the population in Wisconsin. These results are broadly consistent with the results in the main text.
