# Supplementary figures and images for: Forgotten but not gone: A multi-state analysis of modern-day debt imprisonment
Source: PLoS One. 2023 Sep 13;18(9):e0290397. doi: 10.1371/journal.pone.0290397 (PMC10499213; doi:10.1371/journal.pone.0290397)

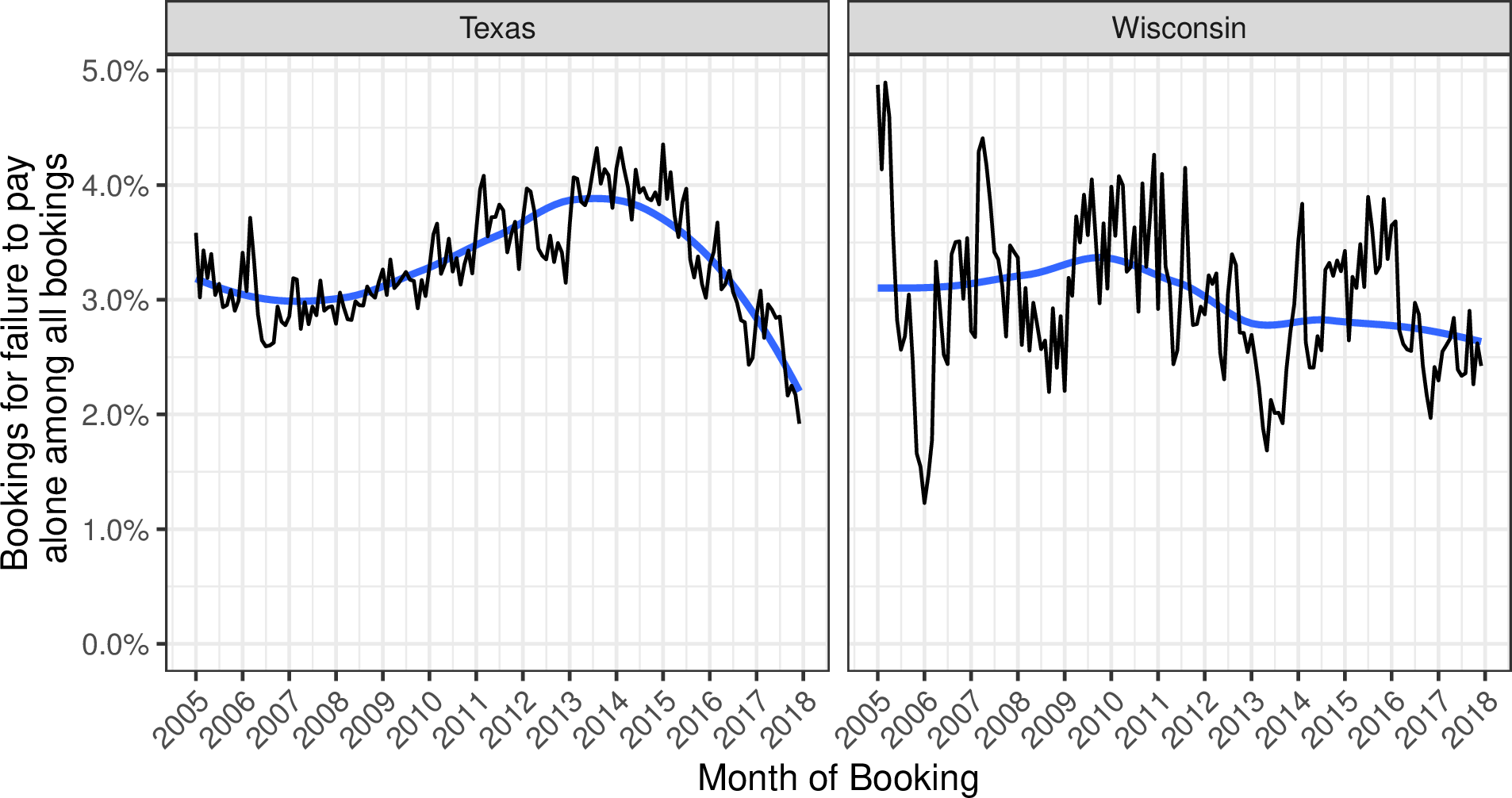

Supplement: S1 Fig — The solid black line indicates the percentage of bookings in the given month across all counties with applicable data in that month. (The counties included in this proportion are the same as in Fig 2, which shows per capita FTP booking rates.) The blue line is a LOESS smoothed average of the percentage of bookings for failure to pay alone. (TIF) [file pone.0290397.s003.tif]

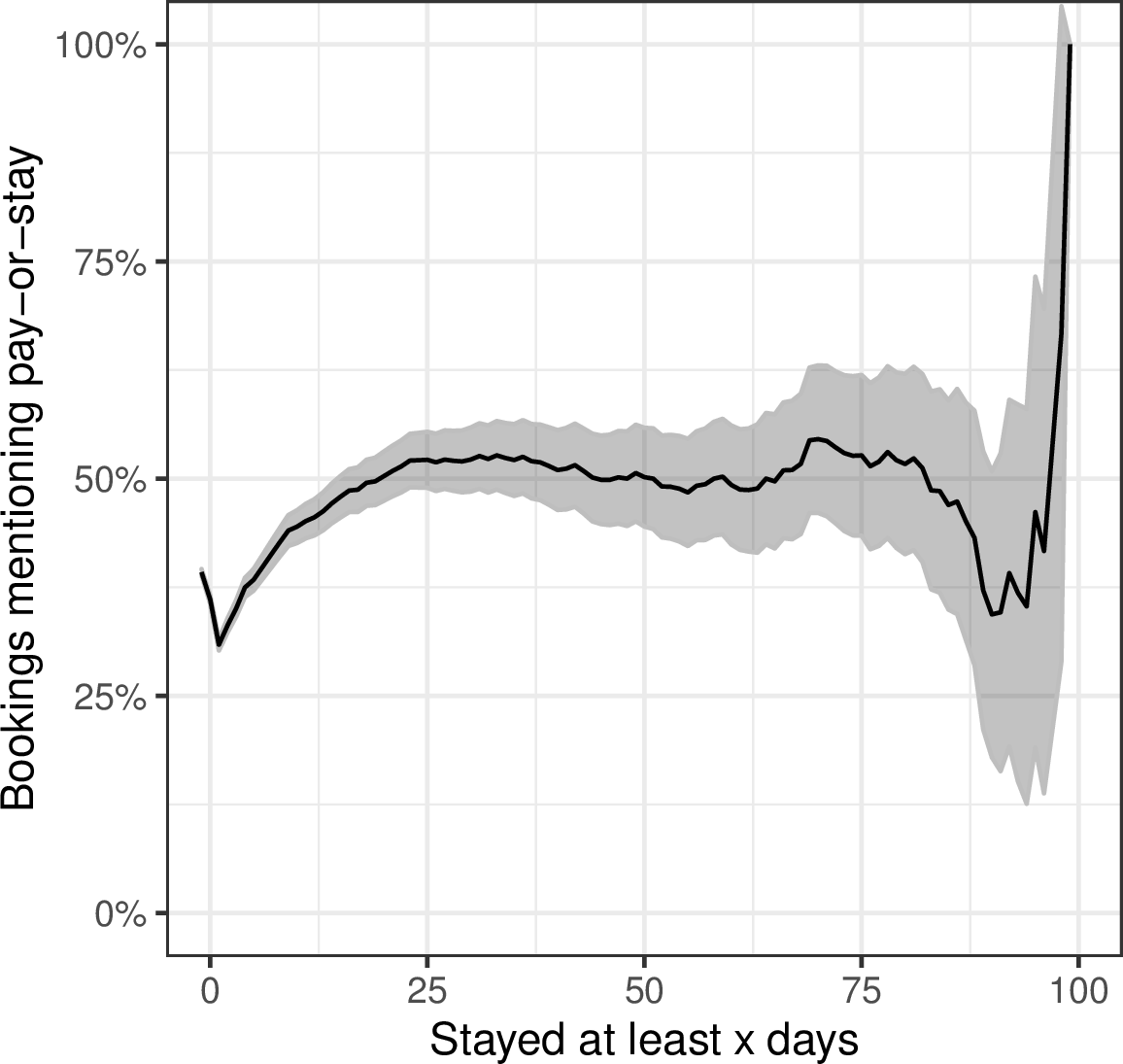

Supplement: S2 Fig — The solid black line represents the percentage of bookings which, based on available information, represents a “pay-or-stay” booking among all bookings for which the length of stay was at least the indicated number of days. Pointwise 95% confidence intervals are shown in gray. We note that the indicated percentages may represent underestimates since a large proportion of bookings comprising our data set do not contain enough detail to confidently determine whether a failure to pay booking was a “pay-or-stay” booking. (TIF) [file pone.0290397.s004.tif]

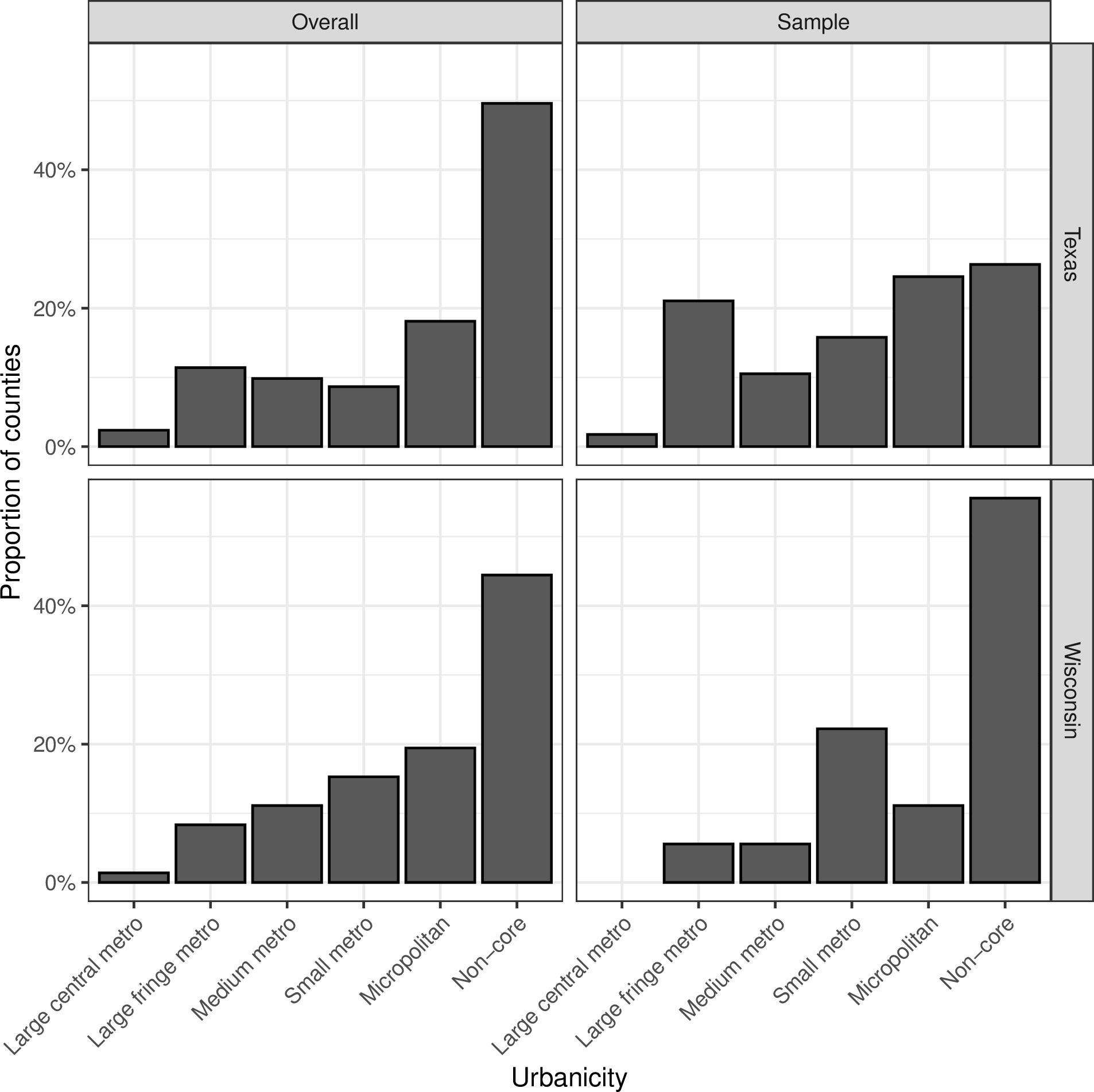

Supplement: S3 Fig — The left-hand panels display the distribution of urbanicity of the counties in Texas and Wisconsin, according to the NCHS Urban-Rural Classification Scheme for Counties [98]. The right-hand panels show the respective distributions among counties present in the sample used to calculate per capita booking rates in the main text. (TIF) [file pone.0290397.s005.tif]

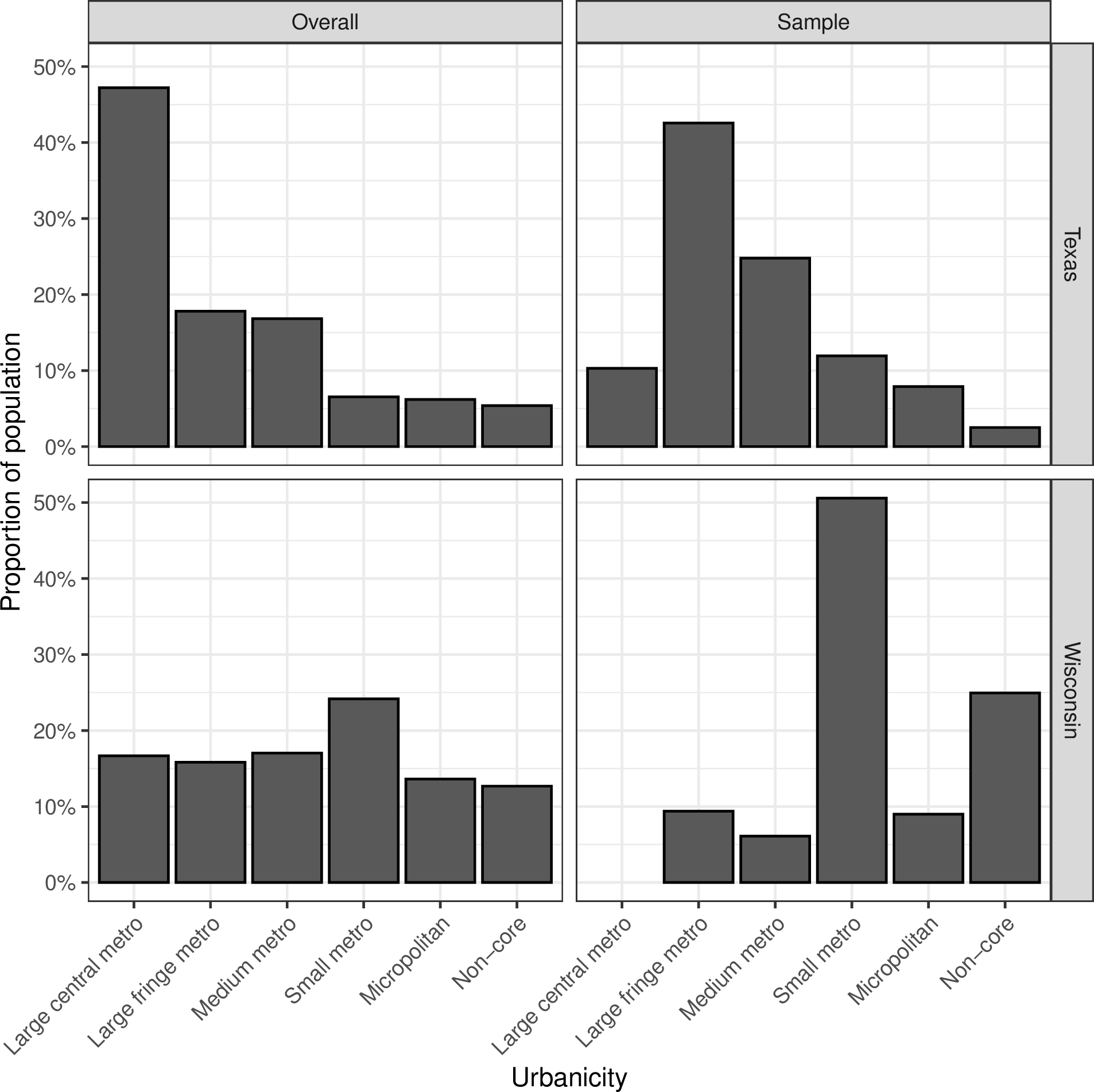

Supplement: S4 Fig — The left-hand panels displays the population-weighted distribution of urbanicity of the counties in Texas and Wisconsin, according to the NCHS Urban-Rural Classification Scheme for Counties [98]. The right-hand panels show the respective distributions among counties present in the sample used to calculate per capita booking rates in the main text, also weighted by population. (TIF) [file pone.0290397.s006.tif]

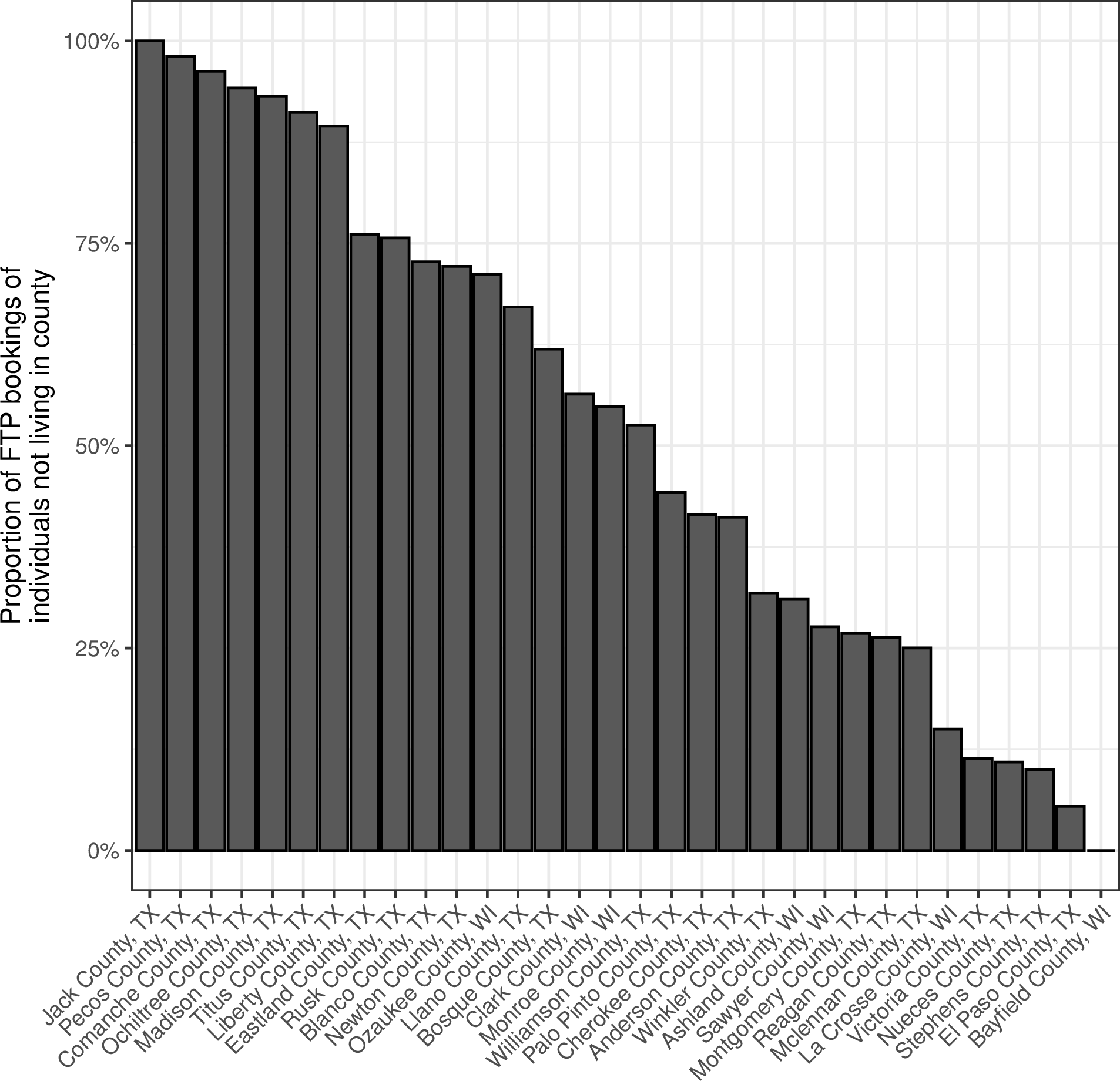

Supplement: S5 Fig — The counties included are those in which ZIP codes were available for the majority of jail bookings. (TIF) [file pone.0290397.s007.tif]
