# Supplementary material for: Forgotten but not gone: A multi-state analysis of modern-day debt imprisonment
Source: PLoS One. 2023 Sep 13;18(9):e0290397. doi: 10.1371/journal.pone.0290397 (PMC10499213; doi:10.1371/journal.pone.0290397)
Supplement: S1 Table — A ‘•’ indicates that the field is present in at least 80% of rows in the indicated county. (PDF) [file pone.0290397.s008.pdf]

State	County	Race	Ethnicity	Sex	Age	ZIP Code	Booking Date	Booking Time	Release Date	Release Time	Charge	Statute	Severity	Release Type	Court	Warrant Type	Bond	Fine	Note	Length of Stay	FTP
Texas	Anderson County	•	•	•		•	•	•			•										•
Texas	Angelina County	•	•	•	•		•	•			•										•
Texas	Austin County	•	•	•	•		•	•			•										•
Texas	Bell County	•	•	•	•		•	•	•	•	•		•	•		•				•	•
Texas	Blanco County	•	•	•	•	•	•		•		•		•	•					•	•	•
Texas	Bosque County	•	•	•	•	•	•		•		•		•	•					•	•	•
Texas	Bowie County	•	•	•	•		•	•			•										•
Texas	Brazoria County	•	•				•	•			•										•
Texas	Brazos County	•	•	•	•		•	•			•								•		•
Texas	Chambers County	•	•	•			•	•			•										•
Texas	Cherokee County	•	•	•	•	•	•		•		•		•	•					•	•	•
Texas	Collin County	•	•	•	•		•	•	•	•	•									•	•
Texas	Comanche County		•		•	•	•		•		•									•	•
Texas	Crane County	•	•	•			•		•		•									•	•
Texas	Denton County	•	•	•			•	•	•	•	•			•						•	•
Texas	Eastland County	•	•	•	•	•	•		•		•		•	•					•	•	•
Texas	El Paso County		•	•		•	•		•		•		•	•						•	•
Texas	Fort Bend County	•	•	•	•		•	•	•				•	•				•		•	•
Texas	Galveston County	•			•		•	•	•	•	•								•	•	•
Texas	Gillespie County	•	•	•			•	•			•										•
Texas	Gray County		•				•		•		•									•	•
Texas	Grayson County	•	•	•			•	•			•										•
Texas	Gregg County	•	•	•			•	•			•								•		•
Texas	Hardin County		•				•														

State	County	Race	Ethnicity	Sex	Age	ZIP Code	Booking Date	Booking Time	Release Date	Release Time	Charge	Statute	Severity	Release Type	Court	Warrant Type	Bond	Fine	Note	Length of Stay	FTP
Texas	Harris County	•		•	•	•	•		•						•	•				•	•
Texas	Hays County	•	•	•	•		•	•			•										•
Texas	Henderson County	•	•	•	•		•	•			•										•
Texas	Hunt County	•	•	•	•		•	•			•										•
Texas	Jack County	•	•	•	•	•	•		•		•		•							•	•
Texas	Jasper County	•	•	•	•		•	•			•										•
Texas	Johnson County		•				•	•			•										•
Texas	Kerr County	•	•	•	•		•	•			•										•
Texas	Lamar County	•	•	•	•		•	•			•										•
Texas	Liberty County	•	•	•	•	•	•		•		•		•	•					•	•	•
Texas	Llano County	•	•	•	•	•	•		•		•		•						•	•	•
Texas	Madison County	•	•	•	•	•	•		•		•		•	•					•	•	•
Texas	Mclennan County			•	•	•	•		•		•			•						•	•
Texas	Montgomery County	•	•	•		•	•		•		•									•	•
Texas	Moore County		•				•		•		•									•	•
Texas	Morris County	•	•	•	•		•	•			•										•
Texas	Navarro County		•				•	•	•	•	•								•	•	•
Texas	Newton County	•	•	•	•	•	•		•		•		•	•					•	•	•
Texas	Nueces County	•	•	•		•	•				•										•
Texas	Ochiltree County	•	•	•	•	•	•		•		•		•	•					•	•	•
Texas	Orange County	•	•	•	•	•	•		•				•			•		•		•	•
Texas	Palo Pinto County	•	•	•	•	•	•		•		•		•						•	•	•
Texas	Pecos County	•	•	•	•	•	•	•			•										•
Texas	Reagan County			•		•	•		•		•	•	•			•				•	•

State	County	Race	Ethnicity	Sex	Age	ZIP Code	Booking Date	Booking Time	Release Date	Release Time	Charge	Statute	Severity	Release Type	Court	Warrant Type	Bond	Fine	Note	Length of Stay	FTP
Texas	Refugio County	•	•	•	•		•	•			•										•
Texas	Rusk County	•	•	•	•	•	•		•		•		•	•					•	•	•
Texas	Scurry County	•	•	•	•		•	•			•										•
Texas	Stephens County	•	•	•	•	•	•		•		•		•	•					•	•	•
Texas	Taylor County		•				•	•			•						•		•		•
Texas	Titus County	•	•	•	•	•	•		•		•		•	•					•	•	•
Texas	Tom Green County	•	•	•	•		•	•			•										•
Texas	Travis County	•		•	•		•		•		•		•		•					•	•
Texas	Tyler County	•	•	•	•		•	•			•										•
Texas	Upshur County	•	•	•	•		•		•		•		•	•					•	•	•
Texas	Victoria County		•			•	•		•		•					•				•	•
Texas	Walker County	•	•	•	•		•	•			•										•
Texas	Webb County	•	•	•			•	•			•						•		•		•
Texas	Wheeler County		•																		
Texas	Williamson County	•	•	•		•	•	•			•										•
Texas	Winkler County	•	•	•		•	•	•			•										•
Wisconsin	Ashland County	•		•	•	•	•		•		•									•	•
Wisconsin	Bayfield County	•		•	•	•	•		•		•	•								•	•
Wisconsin	Brown County		•				•		•		•	•	•							•	•
Wisconsin	Clark County	•	•	•	•	•	•	•	•		•			•						•	•
Wisconsin	Columbia County		•		•		•				•	•							•		•
Wisconsin	Door County		•				•	•			•	•							•		•
Wisconsin	Douglas County		•				•												•		•
Wisconsin	Florence County	•	•	•			•		•					•						•	•

State	County	Race	Ethnicity	Sex	Age	ZIP Code	Booking Date	Booking Time	Release Date	Release Time	Charge	Statute	Severity	Release Type	Court	Warrant Type	Bond	Fine	Note	Length of Stay	FTP
Wisconsin	Fond Du Lac County		•				•		•		•	•								•	•
Wisconsin	Kenosha County	•	•	•	•		•	•	•	•	•	•		•					•	•	•
Wisconsin	Kewaunee County		•								•										•
Wisconsin	La Crosse County	•	•	•	•	•	•	•	•	•	•	•								•	•
Wisconsin	Langlade County		•	•	•		•	•	•		•			•					•	•	•
Wisconsin	Marathon County	•		•			•	•	•		•		•	•					•	•	•
Wisconsin	Marinette County	•	•	•			•		•			•		•						•	•
Wisconsin	Marquette County	•	•			•	•	•			•	•	•		•		•		•		•
Wisconsin	Milwaukee County	•		•	•	•	•	•	•	•	•	•	•	•		•	•		•	•	•
Wisconsin	Monroe County	•	•	•	•	•	•	•	•	•	•	•								•	•
Wisconsin	Ozaukee County	•	•	•	•	•	•	•	•	•		•	•	•	•				•	•	•
Wisconsin	Rusk County	•	•		•		•	•	•	•	•									•	•
Wisconsin	Sawyer County	•		•	•	•	•	•	•	•	•			•						•	•
Wisconsin	Shawano County		•		•		•	•			•	•									•
Wisconsin	Sheboygan County		•				•	•			•	•									•
Wisconsin	Taylor County		•		•		•	•			•	•									•
Wisconsin	Vilas County	•	•	•			•	•	•	•	•	•								•	•
Wisconsin	Walworth County		•								•		•								•
Wisconsin	Waukesha County		•		•		•	•			•	•		•			•		•		•
Wisconsin	Winnebago County	•	•	•	•																
