# Supplementary material for: Forgotten but not gone: A multi-state analysis of modern-day debt imprisonment
Source: PLoS One. 2023 Sep 13;18(9):e0290397. doi: 10.1371/journal.pone.0290397 (PMC10499213; doi:10.1371/journal.pone.0290397)
Supplement: Attachment — Submitted filename: Response to Reviewers.pdf [file pone.0290397.s009.pdf]

Response to Reviewers

PONE-D-23-00711 — *Forgotten but not gone: a multi-state analysis of modern-day debt imprisonment*

Reviewer One

Comment 1. Before providing more suggestions, I want to note that Figure 2 did not appear in the draft that I was asked to review; however, it seems that Appendix Figure S2 is a robustness test for Figure 2. My comments do not account for any specificities of that figure.

> *Figure 2 now appears with the text, along with the robustness check shown in Figure S2.*

Comment 2: My first recommendation would be for the authors to provide more information about what their "jail" variable includes. In particular, I was surprised to see that the median length of stay in both stages, is 1 day. It is my understanding that in some jurisdictions, people can come to jail voluntarily to cancel outstanding warrants; and in the data for such jurisdictions, this could appear as a jail booking. Could this also happen, at least in some counties, for people voluntarily showing up to work on a financial plan with judges? This seems very important to clarify, since it may influence the interpretation of results. This information could likely be obtained by calling courts.

> *The courts we spoke to indicated that they would recall the warrants of an individual who voluntarily came to the courthouse in an effort to clear warrants for unpaid court debts. This is the procedure outlined in the Texas Criminal Code (§ 425.045). However, this does not rule out the possibility that individuals who voluntarily reported directly to jail, rather than the municipal court, to work on a financial plan might, in the jail records, be "booked" without ever actually spending time in jail.*
>
> *Due to differences in the data provided to us, the length of stay is calculated in slightly different ways in different counties. Where only the booking and release dates are available, the length of stay reflects the whole number difference in days between when an individual was booked and released. Where, in addition, booking time and release time are available, length of stay reflects the number of 24 hour periods between booking and release. In the former case, we cannot distinguish between individuals who had short jail stays because they were quickly brought before a judge, and individuals who voluntarily came to jail to clear their warrants but never were actually jailed.*
>
> *In counties where exact times are available, we find that 8% of bookings last an hour or less. While this provides a reasonable upper bound on the proportion of "pro forma" bookings of the kind you describe, we believe most of these are nevertheless probably involuntary. Jail stays on failure-to-pay warrants should, in principle, be short: capias pro fine (CPF) warrants, for instance, require that the arrested individual be brought*

immediately before a judge, at least by the following business day (Tex. Crim. C. § 425.045).

*We have updated the caveats given to our order-of-magnitude estimates in the "*per capita *booking rates" section to reflect this source of uncertainty in our estimates.*

Comment 3: Relatedly, I would recommend that the authors provide a better description of what is means to have a 1-day jail stint -- does this mean 24h, so at least one night in jail? If not, I assume that much of the day was spent waiting in court to meet with a judge. In this case, this paper would be closer to those in Pager et al (2022), who find no effects of court-related fee relief on jail admissions, but do find that it influences future criminal justice contacts.

As detailed in our response to Comment 2, individuals for whom the length of stay is one day spent at least one night in jail. However, as noted above, there are a significant number of individuals who spent less than a day in jail. It's possible that a significant portion of that time was spent in court, depending on what point in the process that individual's book out time reflects. We have clarified this in the "length of stay" section.

Comment 4: On the other side of the distribution, I would have liked to see more details on what drives long (or very long) jail stays -- are these mainly "pay or stay" cases, or what could be driving these very long jail stays? This also seems important to understand, especially from a policy perspective.

Longer stays, compared to short (i.e., 1–3 day) stays, appear to be mainly "pay or stay" cases—the proportion of cases that, for instance, reference "fines paid" or "laid out" as the reason for release is higher than among shorter stays. However, due to the relatively incomplete recordkeeping for a typical case, it is difficult to say for certain that this is the case. We have updated the "length of stay" section with these additional details, and added Figure S4 to the appendix.

Comment 5: In figure 3, I would recommend that the authors plots directly the median and 75th and / or 90th percentiles of length of stay. I would also recommend presenting, in the abstract, median rather than mean length of stay figures -- especially since the high mean is driven by some very long stays (which again, the authors should explain more).

Figure 3 has been modified to include the median, 95th, and 99th percentiles to aid interpretation. The abstract has been updated to present median, rather than mean, length of stay.

Comment 6: I appreciated the inclusion of benchmarks for the scale of jail exposure due to LFOs. A few more comparisons that would be helpful to include, if possible, are: (1) in addition to jail bookings, percent of the jail population at a given point in time incarcerated because of LFOs (which I assume will be quite small, since length of stay is small); and (2) Percent of people with LFOs who are incarcerated. It would also be informative to compute this broken down by racial groups. This second metric may be harder to compute, since it is not clear what the right timeframes to consider LFO issuances is; but for example, if the authors have the date

of the LFO leading to a jail booking, perhaps using other data collected by the Stanford Open Policing Project could help obtain a good initial pool?

> *The percentage of the jail population incarcerated due to LFOs is shown in Figure S3, and is now included in the main text. Calculating the percentage of people with LFOs who have been incarcerated is more difficult. In Texas, at least, the total number of CPF warrants issued across the state is tracked by the Texas Office of Court Administration. Between January, 2012 and December, 2018, Texas municipal and justice of the peace courts issued around 4.4 million CPF warrants. The typical individual jailed for failure to pay in our data had 1.5 CPF warrants, so we can roughly estimate that about 8% of individuals with outstanding CPF warrants were arrested because of their warrants in a given year. Unfortunately, however, the demographics of the individuals for whom CPF warrants were issued are not available, so race-specific rates cannot be calculated without making additional much stronger assumptions.*
>
> *We have updated the text to reflect this figure, along with the caveat that it should be treated as an order-of-magnitude estimate. (In particular, it depends on our estimate of the yearly rate of arrest for failure to pay alone, as well as the fact that individuals who are jailed for unpaid LFOs are representative of the larger population that* has *LFOs, which is likely true only to a coarse degree of approximation.)*

Comment 7: I wasn't sure how informative the results on the distribution of fines was (presented just for Oklahoma), since the sample of people with FTP warrants may be quite different from those incarcerated. For example, I am not sure that the smaller amounts (the average court debt for those with a traffic violation and an FTP warrant is around $500) would lead to a jail booking. I would recommend that the authors try to tie this better to the main results.

> *Unfortunately, since fine information is available for only a relatively small portion of the jail bookings comprising our data set, we cannot directly investigate the fine range that typically leads to jail bookings. Qualitative evidence drawn from the broader debt imprisonment literature suggests, however, that jailings even for relatively small fine amounts are not uncommon. We have updated the text on fine amounts to include this caveat.*

Comment 8: Lastly, I would encourage the authors to engage some more with the recent experimental and quasi-experimental literature seeking to estimate the causal effects of court debt on later outcomes. I think that Pager et al (2022) is the only paper to directly consider jail bookings as an outcome; but for example, Liberman et al (2022) find no or small effects of fines and fees on economic stability. The goal of the present paper is different, and I want to reiterate that I think there is great value in this kind of qualitative work; but tying it better to this literature would help clarify the contribution.

> *We thank the reviewer for their suggestion and list of references. We have updated the discussion as well as the theory section of the introduction. These sections now better reflect the experimental literature on fines and fees, as well as how our*

contribution—descriptive measurement of an important but poorly understood facet of court debt enforcement—fits within this wider line of work.

Reviewer Two

Comment 1: This is a very important issue that is under-studied and requires data that is difficult to collect in a standardized fashion. I commend the authors' efforts to address debt imprisonment. However, I would like a bit more context on existing research and theory on this topic. For example, the authors state that a study was completed in Rhode Island, but provide no details on the study's findings.

> *We have expanded our description of existing research on this topic, including the study of debt imprisonment in Rhode Island, in the section entitled "Criminalization of Poverty."*

Comment 2: I would also like to see a bit more discussion about the rationales for fines and fees: 1) deterrence and 2) to fund local government. The logic of the former is obvious. Regarding the latter, I have read significant anecdotal evidence that units of local government are largely funded on the backs of offenders...particularly in Southern regions of the country. However, is there any systematic analysis of how fines and fees are used directly or indirectly to fund local government? At the end of the document, the authors should spend more time outlining how the current findings support, reject, or expand upon these rationales. As a side note, I constantly hear the debate between the importance of deterrence versus rehabilitation concerning more serious crimes, but we never talk about rehabilitation when it comes to minor crimes and traffic violations that result in fines and fees.

> *While the nature of our data does not allow us to comment on this subject directly, in the theory section of the introduction and in the discussion, we have expanded our discussion of the rationales for fines and fees, including references to the growing literature addressing this question.*

Comment 3: I would like more details on how county data in "raw formats" was assessed and cleaned to develop the data sets. My experience is that this is an incredibly challenging task and I believe it would behoove the broader field of research in criminal justice and corrections to collaborate on how we address this issue.

> *Addressing the problem of the multiplicity of formats in which data were stored and provided to us was one of the most challenging aspects of this research. In the "Datasets" section, we now provide additional details on how we standardized data across formats, as well as pointing readers to the codebase we developed for this project, where the tools we developed to solve the data integration problems we encountered are freely available to other researchers.*

Comment 4: The authors indicate that data from city jails might be omitted. Does this mean that rural areas were more likely to have their data included in the data sets? Our experience is that data from rural areas is much less automated than data from urban jurisdictions. Was this the case in the current data collection? If so, how was this addressed? My anecdotal knowledge is that rural jurisdictions rely more on fines and fees for their budgets, as compared to more urban

jurisdictions. Do the current findings support that assertion? In short, I would like more information about the rural/urban makeup of the data sets, rural/urban differences in data collection, and urban/rural differences in findings.

> *We have added Figure S7 to the appendix, which compares the urbanicity of the counties we studied to the urbanicity of the states as a whole. In general, we found it easier to obtain records from large, urban counties than smaller rural counties where digital record keeping systems were usually in place. We aggressively pursued jail records from smaller counties, but, due to the logistical constraints, our coverage for rural areas is less than for urban areas. We have updated the text to clarify these limitations. (For avoidance of doubt, we obtained data from county jails in both rural and larger urban counties; the so-called "city jails" or "municipal lockups" from which we did not obtain data are smaller facilities not under the jurisdiction of the county sheriff or Texas Commission on Jail Standards. We have added this additional context to the main text.)*
>
> *Unfortunately, because very few jurisdictions provided us with actual fine amounts, our data do not speak directly to whether rural jurisdictions are more reliant on fines than urban jurisdictions. However, this question has received increasing interest in the literature, and we have expanded our discussion of this in the discussion and introductory theory section, as noted above.*

Comment 5: The "denominator problem" is a serious problem for looking at racial/ethnic disparities in traffic stops. It is also a major issue for this analysis as those receiving traffic violations might be "passing through" rather than live in the jurisdiction of study. Are you able to analyze the data separately for cases that are or are not related to traffic violations to see if there are changes in the racial/ethnic disparities?

> *Rather than comparing traffic to non-traffic cases, in an effort to directly address this aspect of the denominator problem, we have added an additional analysis to the appendix looking at racial disparities where we exclude individuals who do not live in the county in question based on ZIP code. Our findings under this alternative methodology are broadly consistent with the findings presented in the main text. See Appendix S4.*

Comment 6: Finally, and related to my earlier point about deterrence and rehabilitation, for whatever reason, speeding has become a more serious problem in my state of residence. The State Patrol has seen huge increases in traffic violations for individuals traveling over 100 mph, causing serious concerns for traffic safety. Fines are our primary mechanism to attempt to deter speeders. Although I agree that fines are problematic to the extent that the lead to debt imprisonment, what is an alternative for deterring traffic violations? In my criminology courses, I have introduced Scandinavian methods of adopting progressive fines for speeding, such that rich people could be fined thousands of dollars for a simple speeding violation (which blows the minds of students). High fines on the rich can subsidize smaller fines on the poor. I would be interested in seeing more proposed solutions to the issues addressed in this paper.

> *We have updated the discussion to better reflect our views on how policy makers should balance the objectives of achieving traffic safety as well as reducing debt imprisonment.*

We thank the reviewer for incorporating progressive fines into our discussion. We also detail other avenues for achieving traffic safety objectives that don't engender the same issues as the status quo, such as traffic calming measures, non-monetary penalties, and direct targeting of traffic safety in enforcement.
